# Mouse Genetics Suggests Cell-Context Dependency for Myc-Regulated Metabolic Enzymes during Tumorigenesis

**DOI:** 10.1371/journal.pgen.1002573

**Published:** 2012-03-15

**Authors:** Lisa M. Nilsson, Tacha Zi Plym Forshell, Sara Rimpi, Christiane Kreutzer, Walter Pretsch, Georg W. Bornkamm, Jonas A. Nilsson

**Affiliations:** 1 Department of Molecular Biology, Umeå University, Umeå, Sweden; 2 Sahlgrenska Cancer Center, University of Gothenburg, Gothenburg, Sweden; 3 Institute of Human Genetics, Helmholtz Zentrum München, National Research Center for Environmental Health, München, Germany; 4 Institute of Clinical Molecular Biology and Tumour Genetics, Helmholtz Zentrum München, National Research Center for Environmental Health, München, Germany; Fred Hutchinson Cancer Research Center, United States of America

## Abstract

c-Myc (hereafter called Myc) belongs to a family of transcription factors that regulates cell growth, cell proliferation, and differentiation. Myc initiates the transcription of a large cast of genes involved in cell growth by stimulating metabolism and protein synthesis. Some of these, like those involved in glycolysis, may be part of the Warburg effect, which is defined as increased glucose uptake and lactate production in the presence of adequate oxygen supply. In this study, we have taken a mouse-genetics approach to challenge the role of select Myc-regulated metabolic enzymes in tumorigenesis *in vivo*. By breeding λ-*Myc* transgenic mice, *Apc* ^Min^ mice, and *p53* knockout mice with mouse models carrying inactivating alleles of *Lactate dehydrogenase A* (*Ldha*), *3-Phosphoglycerate dehydrogenase* (*Phgdh*) and *Serine hydroxymethyltransferase 1* (*Shmt1*), we obtained offspring that were monitored for tumor development. Very surprisingly, we found that these genes are dispensable for tumorigenesis in these genetic settings. However, experiments in fibroblasts and colon carcinoma cells expressing oncogenic Ras show that these cells are sensitive to *Ldha* knockdown. Our genetic models reveal cell context dependency and a remarkable ability of tumor cells to adapt to alterations in critical metabolic pathways. Thus, to achieve clinical success, it will be of importance to correctly stratify patients and to find synthetic lethal combinations of inhibitors targeting metabolic enzymes.

## Author Summary

Cancer occurs when cells change their behavior and start to divide in an uncontrolled manner. To achieve this altered behavior, cells need to change their metabolism to be able to grow even when nutrient and oxygen supplies are limiting. Therefore, targeting metabolic pathways could be used to treat patients suffering from cancer. Here we studied a gene called *MYC*, which can regulate many metabolic pathways. By using genetically modified mice we can show that tumors have a remarkable ability to change their metabolism, even if key enzymes are removed. Taken together, our data suggest that metabolic disturbance by drugs in the clinic may present a future challenge.

## Introduction

Activation of one of the three *MYC* oncogenes is frequently selected for during tumorigenesis. These genes encode the transcription factors c-Myc, N-Myc and L-Myc that regulate a large number of downstream target genes. Although most of the work on *MYC* oncogenes has involved their role in cell proliferation, it is becoming clear that they may be involved in most aspects of oncogenic transformation [1]. As such, unravelling the mechanisms by which Myc proteins activate genes, and which are the essential genes, is paramount as studies resolving these mechanisms may open up new avenues of targeted intervention against various cancers.

Some of Myc's earliest discovered transcriptional targets were genes encoding metabolic enzymes such as *Ornithine decarboxylase* [Odc] [2], [3], *Lactate dehydrogenase A* [Ldha] [4] and *Carbamoyl-phosphate synthase/aspartate carbamoyltransferase/dihydroorotase* [Cad] [5]. Later studies using expression profiling identified even more of these genes, indicating that Myc is a master regulator of cellular metabolism and cell growth [6], [7]. Interestingly, inhibition of polyamine biosynthetic enzymes Odc and Spermidine synthase have shown efficacy in chemoprevention of several cancers in experimental models [8]–[14] and in colon cancer patients [15]. Furthermore, Myc-regulated Ldha, Pyruvate kinase M2 and Glutaminase have also emerged as promising targets based on experimental models of human cancer [16]–[23], suggesting that targeting various metabolic pathways regulated by Myc may prove beneficial in cancer therapies of patients. To gain *in vivo* support for this notion we performed genetic ablation experiments in mice to determine the individual contribution to tumorigenesis of three different Myc-regulated metabolic enzymes.

## Results

**Figure 1:**
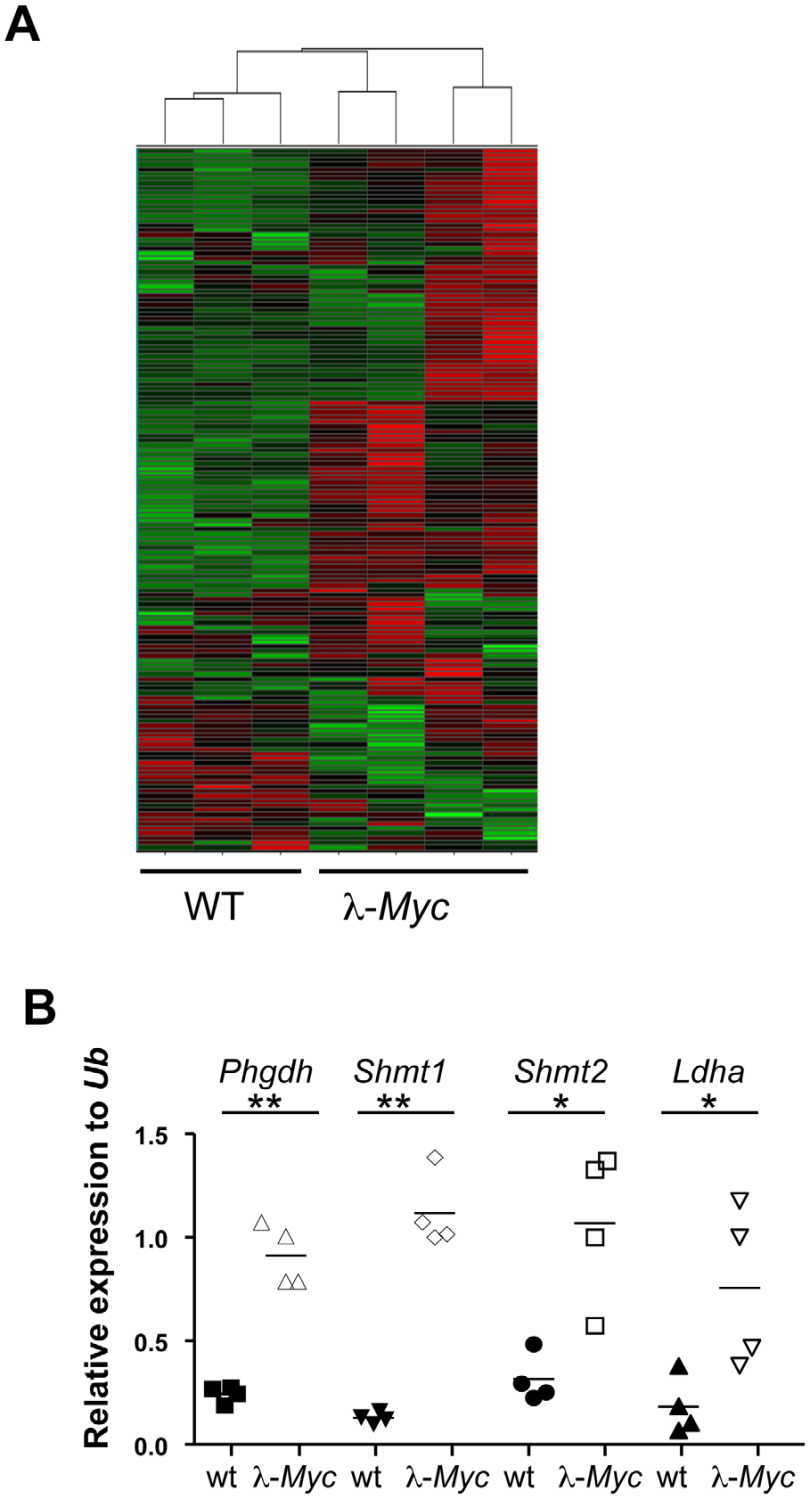
Myc regulates the metabolic transcriptome. (A) Unsupervised hierarchical clustering of Illumina bead arrays made from RNA of splenic B cells from three wildtype and four precancerous λ-*Myc* transgenic mice. See Table S1 for genes used in the clustering. (B) qRT-PCR confirmation of 4 of the 20 most significantly, or most elevated expressed genes, in B220-sorted B cells from λ-*Myc* transgenic mice. *indicates p<0.05 and ** indicates p<0.01.

To identify critical Myc-regulated metabolic enzymes, we performed Illumina bead chip arrays on RNA isolated from 4–6 week old wildtype or precancerous, B cell lymphoma-prone λ-*Myc* transgenic mice, where the human *MYC* gene is under the control of the immunoglobulin (Ig) λ enhancer [24]. Interestingly, when we performed unsupervised Hierarchical clustering on 153 genes (Table S1) encoding metabolic enzymes involved in glycolysis, the Kreb's cycle, oxidative phosphorylation, serine synthesis and one-carbon metabolism, all expression profiles from *Myc*-transgenic B cells grouped together despite some intra-individual expression level differences that could be due to expression levels of *MYC* and developmental stage of the B-cell compartment (Figure 1A). Largely, these data are supportive of the Myc target gene database (http://www.myccancergene.org).

Many tumor cells use aerobic glycolysis, producing lactate even in the presence of oxygen (the Warburg effect) [25]. Most of the glucose from the enhanced glucose uptake is however used to provide metabolites for anabolic processes, such as fat and nucleotide synthesis. Glycolysis and nucleotide metabolism are linked at several steps including the pentose phosphate shunt and via the phosphorylated pathway of serine synthesis (Figure S1). In the latter, 3-phosphoglycerate dehydrogenase (Phgdh) catalyzes the first step and serine hydroxymethyltransferases (Shmt1 and Shmt2) use the final product to produce folate metabolites that are critical for several metabolic pathways including methylation and thymidylate synthesis. Given that Myc regulates genes involved in many metabolic pathways we decided to focus on genes that were induced by Myc and for which there were genetic tools accessible at the start of this project. qRT-PCR analysis confirmed that the selected genes had elevated expression in *Myc*-transgenic B cells (Figure 1B).

Shmt1 and Shmt2 protein levels and their combined activity are elevated in B cells from λ-*Myc* transgenic mice (Figure S2A and S2B). So to analyze the role of Shmt1 in Myc-induced tumorigenesis, we obtained embryonic stem (ES) cells carrying a gene-trapped allele of *Shmt1*, which were injected into blastocysts to generate chimeric mice. The offspring from these mice generated wildtype, heterozygous or homozygous *Shmt1* knockout mice at the expected Mendelian frequency (Figure S2C and S2D), corroborating a recent publication reporting that Shmt1 is dispensable for mouse development [26].

**Figure 2:**
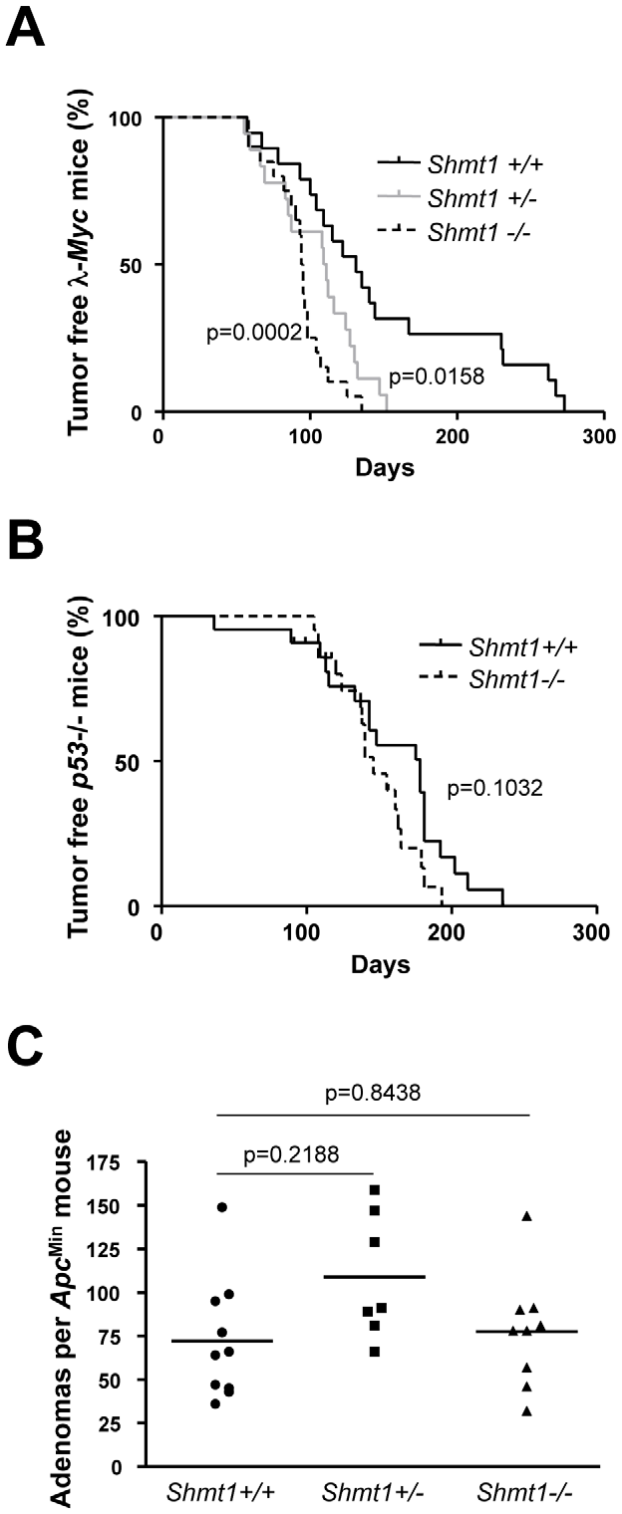
*Shmt1* loss accelerates lymphomagenesis in λ-*Myc* transgenic mice. (A) Survival curve of λ-*Myc* mice generated from interbreedings between *Shmt1* knockout and λ-*Myc* transgenic mice. λ-*Myc*; *Shmt1* ^+/+^ n = 19, λ-*Myc*; *Shmt1* ^+/−^ n = 18, λ-*Myc*; *Shmt1* ^−/−^ n = 20. (B) Survival curve of *p53* knockout mice of the indicated *Shmt1* genotypes. *p53* ^−/−^; *Shmt1* ^+/+^ n = 22, *p53* ^−/−^; *Shmt1* ^−/−^ n = 21. (C) Amount of adenomas in *Apc* ^Min^ mice with different *Shmt1* genotypes.

The homozygous *Shmt1* mutant mice did not express any Shmt1 protein in the tissues analyzed (Figure S2E) making them suitable for the assessment of the role of this gene for Myc-induced tumorigenesis. To that end, we first back-crossed the *Shmt1* knockout mouse for 10 generations to the C57BL/6 strain and then the interbred it with 3 different tumor models where *Myc* is either the direct cause of transformation (λ-*Myc* transgenic mice, Figure 2A [24]) or constitutes an important circuit (*p53* knockout mice, Figure 2B [27] and *Apc* ^Min^ mice of intestinal tumorigenesis, Figure 2C [28]). Surprisingly, we did not observe any major negative impact of *Shmt1* loss on tumor initiation and development in these models. The only significant effect was a clear acceleration of disease in the λ-*Myc* transgenic mice (Figure 2A), suggesting a B-cell specific event. Taken together these data argue against Shmt1 as a target for chemotherapy.

Serine and folate metabolites can also be made via pathways involving Shmt2 and Phgdh. However, to assess the role of Shmt2, we were forced to take a different approach, as *Shmt2* gene-trap clones or knockout mice were not available when initiating this project. We hence infected Colon 26 cells, which carry an NMU-induced *Kras* mutation [29], with lentiviruses expressing shRNA directed against Shmt2 and Phgdh. Despite achieving potent knockdown levels, we did not observe any effect on viability or ability to form subcutaneous tumors when injected into syngenic Balb/c mice, as compared to cells infected with a control lentivirus (Figure S3A and S3B).

**Figure 3:**
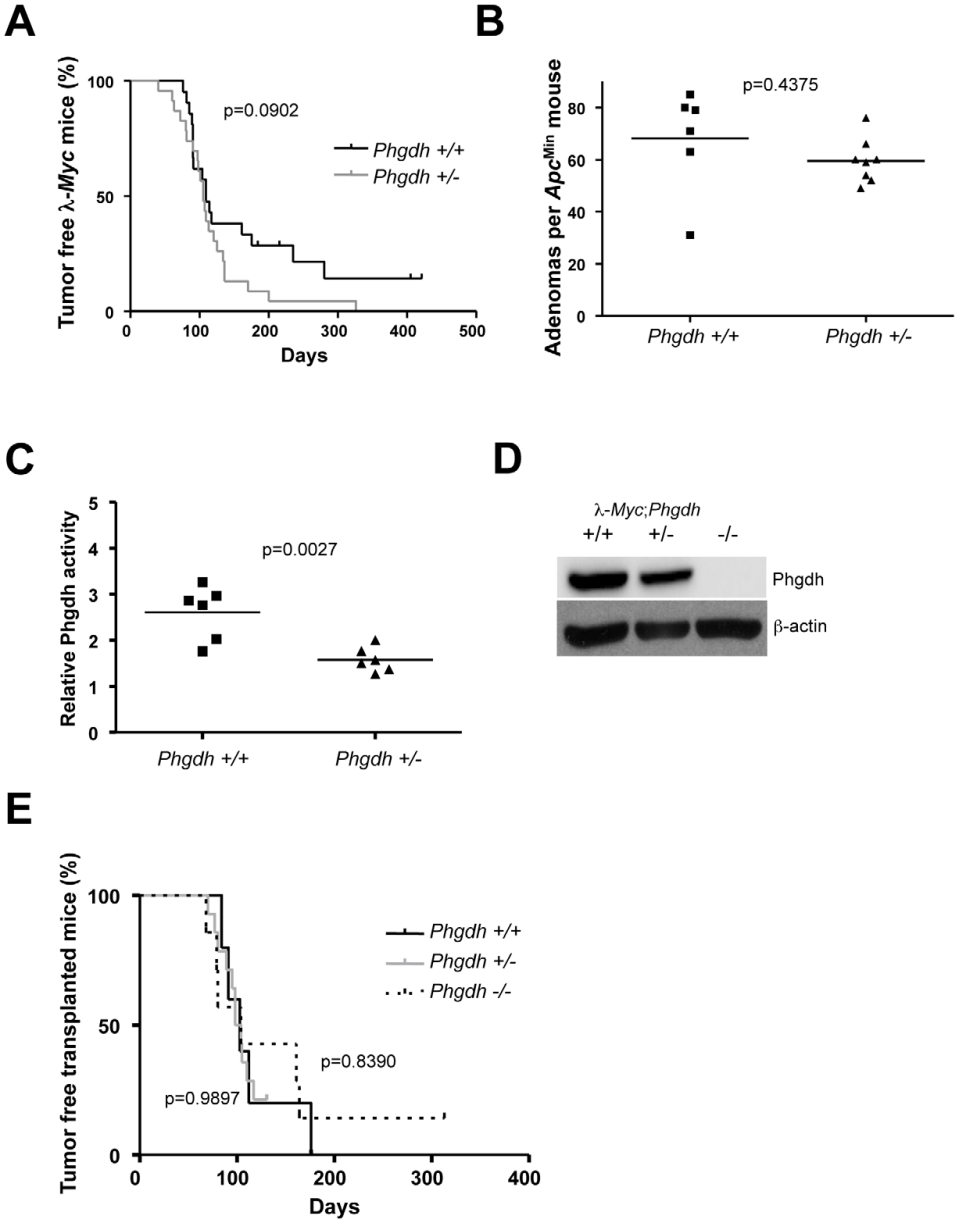
Phgdh is dispensable for lymphomagenesis in λ-*Myc* transgenic mice. (A) Survival curve of λ-*Myc* mice generated from interbreedings between *Phgdh* ^+/−^ and λ-*Myc* transgenic mice. λ-*Myc*; *Phgdh* ^+/+^ n = 21, λ-*Myc*; *Phgdh* ^+/−^ n = 23. (B) Amount of adenomas in *Apc* ^Min^ mice with different *Phgdh* genotypes. (C) Enzymatic activity of Phgdh analyzed in six λ-*Myc*; *Phgdh* ^+/+^ and in six λ-*Myc*; *Phgdh* ^+/−^ lymphomas. (D) Western blot analysis confirming that Phgdh is absent in tumors arising in recipient mice from λ-*Myc;Phgdh* ^−/−^ embryos. (E) Survival curve of C57BL/6 mice transplanted with l-*Myc* transgenic E13.5 FLC of indicated *Phgdh* genotype. λ-*Myc*; *Phgdh* ^+/+^ n = 5, λ-*Myc*; *Phgdh* ^+/−^ n = 14, λ-*Myc*; *Phgdh* ^−/−^ n = 7.

To further assess the effect of Phgdh loss in different tumor models, we obtained a *Phgdh* knockout mouse. Since Phgdh is essential for neurogenesis [30], *Phgdh* null embryos die at around embryonic day (E) 13.5, which prevented us from analyzing the effects of loss of *Phgdh* by conventional breeding to our tumor models. We therefore started out by assessing the impact of removal of just one allele of *Phgdh* on tumorigenesis in λ-*Myc* transgenic mice and in *Apc* ^Min^ mice. At variance with *Odc*, which is haploinsufficient for tumor progression in these models, *Phgdh* heterozygosity did not impact tumorigenesis in these tumor models (Figure 3A and 3B), despite the 50% reduction in Phgdh activity (Figure 3C). As an alternative approach, we crossed λ-*Myc*; *Phgdh^+/−^* mice with *Phgdh^+/−^* mice and isolated hematopoietic stem cells from E13.5 fetal livers. These cells were then used to reconstitute lethally irradiated syngenic recipients, creating lymphoma-prone mice with varying expression of Phgdh (Figure 3D). Even in this setting, Phgdh was dispensable for Myc-induced tumorigenesis (Figure 3E), suggesting that hematopoiesis and Myc-driven tumorigenesis can occur in the absence of Phgdh.

**Figure 4:**
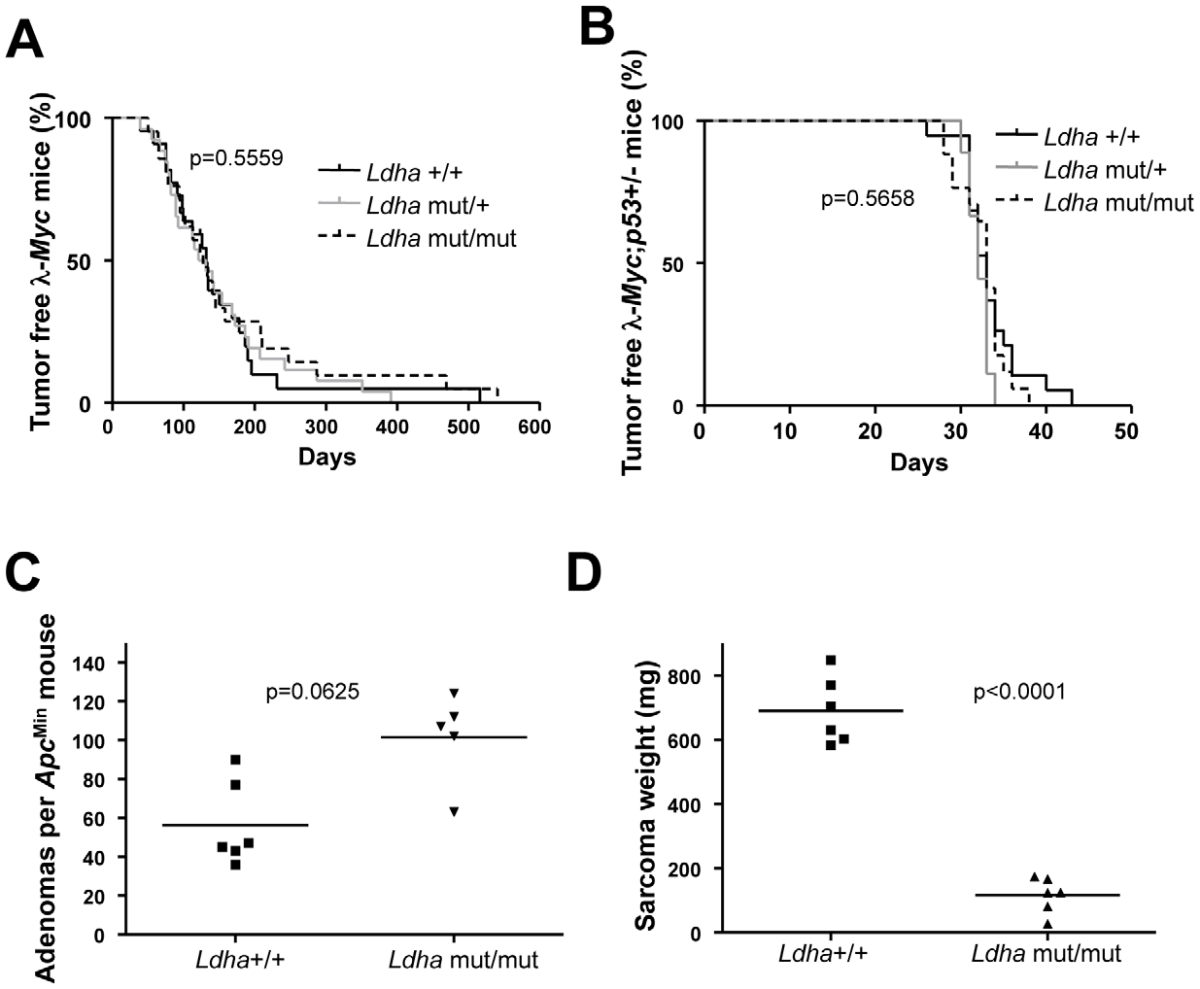
*Ldha* is dispensable for Myc-induced lymphomagenesis but not for the development of Ras-induced fibrosarcomas. (A) Survival curve of λ-*Myc* mice generated from interbreedings between *Ldha* ^mut/mut^ and l-*Myc* transgenic mice. λ-*Myc*;*Ldha* ^+/+^ n = 13, λ-*Myc*;*Ldha* ^+/mut^ n = 26, λ-*Myc*;*Ldha* ^mut/mut^ n = 21. The p-value is derived from comparing the survival of λ-*Myc*;*Ldha* ^+/+^ to λ-*Myc*;*Ldha* ^mut/mut^ mice. (B) Survival curve of λ-*Myc* mice generated from interbreedings between *Ldha* ^mut/mut^, *p53* knockout and λ-*Myc* transgenic mice. λ-*Myc*;*p53* ^+/−^;*Ldha* ^+/+^ n = 19, λ-*Myc*;*p53* ^+/−^;*Ldha* ^+/mut^ n = 9, λ-*Myc*;*p53* ^+/−^;*Ldha* ^mut/mut^ n = 17. The p-value is derived from comparing the survival of λ-*Myc*;*p53* ^+/−^;*Ldha* ^+/+^ to λ-*Myc*; *p53* ^+/^;*Ldha* ^mut/mut^ mice. (C) Amount of adenomas in *Apc* ^Min^ mice with different *Ldha* genotypes. (D) MEFs of different *Ldha* genotypes were infected with oncogenic Ras. After subcutaneous injection into syngenic C57BL/6 mice, tumor weights were monitored 16 days post-injection. Two mice were injected with cells per *Ldha* genotype and the experiment was repeated with three different MEF isolates per *Ldha* genotype.

Phgdh is linked to glycolysis and could potentially divert metabolites away from pyruvate usage in the TCA cycle in the mitochondrion. Pyruvate is also kept from entering the TCA cycle via the action of Ldha, encoded by another Myc-regulated gene [23]. Except for RNAi or antisense studies in established tumors, it is not known whether Ldha is needed for the actual transformation event *in vivo*. To assess this we used a mouse model carrying a procarbazine-induced homozygous germline mutation of *Ldha* [31]. The mutation has been mapped to an aspartate 223 to histidine exchange which results in a very strong phenotype in erythrocytes causing anemia that is counteracted by extra-medullary hematopoiesis with an associated splenomegaly (Figure S4A). We crossed *Ldha* mutant mice with λ-*Myc* mice to generate mice of all relevant genotypes. Some of these mice were sacrificed before they developed tumors to allow analysis of Ldh activity in splenic B cells. Other mice were aged and monitored for tumor development. As seen in Figure S4B, splenic B cells from λ-*Myc* mice exhibited an elevated level of Ldh activity - consistent with the expression analysis in Figure 1 – whereas the *Ldha* mutation severely diminished Ldh activity in B cells from both non-transgenic and Myc transgenic mice (Figure S4B). Unexpectedly, the *Ldha* mutation did not affect Myc-induced B-cell lymphomagenesis. In two independent survival curves generated at Umeå University and Helmholtz Center Munich the median survival time for λ-*Myc*;*Ldha* ^mut/mut^ was similar to that of λ-*Myc*;*Ldha* ^wt^, with no statistical difference (Figure 4A for the Umeå-generated survival curve; Munich curve is shown in Figure S4C).

Our unexpected results suggest several possibilities: either Ldha is dispensable for Myc-induced lymphomagenesis; or a compensatory mechanism is occuring; or Ldha deficiency alters the route of transformation. Firstly, a compensation by another Ldh form is improbable since Ldh activity and Ldhb expression were very low or absent in tumors arising in λ-*Myc*;*Ldha* ^mut/mut^ mice (Figure S4D and S4E). Secondly, Myc-induced lymphomagenesis in the mouse is known to involve spontaneously arising, cooperating oncogenic mutations of tumor suppressors and other oncogenes to block the oncogenic stress response of Myc [32], [33]. Hence, overexpression of anti-apoptotic proteins such as Bcl-2 or genetic deletion of one tumor suppressor allele such as Arf or p53 dramatically accelerates lymphomagenesis [34]–[36]. To neutralize the genetic heterogeneity in the cooperating oncogenic lesion during lymphomagenesis we interbred the *p53* knockout mouse with the *Ldha* mutant mouse and the λ-*Myc* mouse. λ-*Myc*;wildtype, λ-*Myc;Ldha* ^mut/wt^ or λ-*Myc;Ldha* ^mut/mut^ were made heterozygous for *p53* by interbreeding. All mice developed disease at an accelerated rate as compared to λ-*Myc* mice (Figure 4B). The tumors that developed lost the wildtype *p53* allele (data not shown). Moreover, the frequency of *p53* mutation in the tumors that developed in the first cross (Figure 4A) was not different between *Ldha* genotypes (data not shown). We conclude that Myc-induced lymphomagenesis can occur normally in mice lacking fully functional Ldha.

Studies using antisense or RNAi have shown that Ldha is important for breast carcinoma, neuroblastoma, fumarase-deficient renal cell cancers, as well as fibroblast and B-cell tumor cells *in vitro* [17], [18], [20], [23], [37]. Although the specific combination of Myc overexpression with loss of p53 was previously unexplored, other explanations to the differences between our findings and those of others, like experimental methods, culture conditions, oxygen supply or oncogenic pathway, could be at play. To test if a dependency of Ldha could be revealed in settings where Myc is downstream rather than the primary oncogenic instigator, we interbred *Apc* ^Min^ mice and *p53* knockout mice with the *Ldha* mutant mouse. As seen in Figure 4C, Ldha deficiency did not impact adenomagenesis in the *Apc* ^Min^ mice. However, we also created *p53* ^−/−^;*Ldha* ^mut/mut^ or *Ldha* ^wt^ mouse embryo fibroblasts (MEFs) which were transduced with an oncogenic H-Ras (pBabe-Hras^G12V^-puro) retrovirus. Interestingly, whereas no growth defect could be observed *in vitro*, the *Ldha* mutant Ras-transformed *p53* knockout MEFs generated significantly smaller tumors *in vivo* that appeared less vascularized (Figure 4D and Figure S5A). This effect was not a result of varying expression of oncogenic H-Ras in the different tumors (Figure S5B). Moreover, Colon 26 cells with an endogenous oncogenic *Kras* allele could not be propagated when transduced with lentiviruses expressing two different *Ldha* shRNAs despite the fact that these constructs were not lethal in NIH 3T3 cells (data not shown). To confirm the dependency on Ldha we also co-transfected a GFP expressing plasmid with the lentiviral expression constructs expressing *Ldha* shRNA into Colon 26. As seen in Figure S3C and S3D, we were able to knockdown expression of Ldha in the cells, which resulted in a progressive loss of cells from 48 h post-transfection.

To investigate the ability of *Ldha*-deficient fibroblasts to proliferate in a hypoxic environment, NIH 3T3 cells infected with Myc (pWLBlast-c-*Myc*) or Ras (pBabe-Hras^G12V^-hygro) retroviruses and control or *Ldha* shRNA lentiviruses were exposed to hypoxia. As expected, hypoxia resulted in the induction of Ldha and Pdk1, both downstream targets of Hif1α (Figure S5C). Interestingly, cells infected with the *Ldha* shRNA incorporated less 3H-thymidine than cells infected with a control lentivirus (Figure S5D). Thus, Ldha is required under defined conditions such as hypoxia and/or in cells with a deregulated Ras pathway. Therefore an Ldha dependency may not be manifested in a Myc-induced lymphomagenesis setting.

In agreement with this notion, the λ820 mouse B-cell lymphoma line established from λ-*Myc* mice [38] succumbed to apoptosis when exposed to hypoxia (Figure S6A and S6B), regardless of whether or not *Ldha* was knocked down (Figure S6C). In addition, *Ldha* knockdown did not impact lymphomagenesis *in vivo* (Figure S6D), although knockdown still left a substantial amount of *Ldha* transcript and activity in this highly Ldha-expressing cell line (Figure S6C and S6E). Nevertheless, given the sensitivity of λ-*Myc* lymphoma cells to hypoxia (Figure S6A and S6B), it is unlikely that tumorigenesis in this model contains a hypoxic component and thereby dependency on elevated Ldha activity. Indeed, immunohistochemistry showed that *Ldha* wildtype or mutant lymphomas from λ-*Myc* mice exhibited a remarkable sparse expression of angiogenic markers CD34 and SMA (Figure S7A). Despite this there were no signs of obvious necrotic areas, suggesting that nutrients and oxygen can diffuse in these non-solid tumors. The staining results were not due to non-functional antibodies as they readily detected the angiogenic markers in normal spleen and in lymphomas that had disseminated in spleens of λ-*Myc* mice (Figure S7B). It therefore appears as if lymphomas arising in lymph nodes of λ-*Myc* mice are neither angiogenic, hypoxic or dependent on Ldha activity.

## Discussion

We are today beginning to appreciate the fact that oncogenes and tumor suppressor genes not only regulate cell proliferation, immortalization, apoptosis, metastasis and angiogenesis [39] but also cellular metabolism. The change in metabolism and the Warburg effect were for a long time believed to be self-evident and secondary to transformation. It is now known that the metabolic changes occur simultaneously and are governed by the same signal transduction pathways as those governing cell proliferation [40]. Since different tumor cells transform in response to variations of oncogenic mutations it is therefore likely that tumor cells can, or even have to, make different metabolic adaptations as well. Some of these adaptations may make the cells dependent on a certain enzyme, whereas others do not. Our study highlights the Ras oncogene as a potential pathway that requires Ldha, illustrated in Ras-transformed fibroblasts and Colon 26 cells, which carry an endogenous *Kras* mutation. Indeed, it has previously been shown that *neu*-transformed breast cancer cells are sensitive to Ldha inhibition by RNAi [37]. Since these cells have an activated Ras pathway [41], this may explain why knockdown of *Ldha* sensitizes these cells. The potential explanation why Ras-induced fibrosarcomas are sensitive to the *Ldha* mutation *in vivo* is that they are not inherently angiogenic, making them sensitive to metabolic perturbation before angiogenesis has occurred. The fact that Myc can stimulate angiogenesis independently of hypoxia-inducible factors [42]–[44] may account for the lack of impact on lymphomagenesis upon mutation of e.g. *Ldha*. Indeed, we show here that lymphomas from λ-*Myc* mice are very sensitive to hypoxia, most likely since they are Myc-driven and therefore rely on the TCA cycle and oxidative phosphorylation [45].

Folate biosynthesis has been linked to cancer both from studies on dietary supplements and by the identification of polymorphisms in genes encoding enzymes in folate biosynthesis like *SHMT1*, *MTHFR* and *TS* [46]. In addition, certain drugs like methotrexate target the folate biosynthetic pathway suggesting that this pathway is of critical importance for tumor cell survival. Interestingly, *Shmt2* was first identified as a target in a screen for genes that can rescue the growth defect of *Myc* null rat fibroblasts [47]. In the same study *Shmt1* was also shown to be a Myc transcriptional target gene but it was not further functionally characterized. Herein, we provide evidence that *Shmt1* is dispensable for Myc-induced lymphomagenesis and that its deletion even accelerates tumorigenesis. The reason for this acceleration is unknown. It could involve effects on senescence or B-cell development as deletion of genes like *Suv39h1* and *E2f2* accelerates tumorigenesis by these mechanisms, respectively [48], [49]. However, our data corroborate other very recent studies. Using an *Shmt1* knockout mouse [50], the Stover group showed that deletion of one allele of *Shmt1* promotes adenomagenesis in the *Apc* ^Min^ mice when administered a special diet [26]. In our study the *Shmt1* heterozygous *Apc* ^Min^ mice also have the largest mean amount of adenomas, albeit we did not investigate the impact of diet on this model. Interestingly, homozygous deletion of *Shmt1* did not impact adenoma formation in *Apc* ^Min^ mice since there was a compensatory increase in thymidylate kinase (TK1) expression [47]: a salvage pathway for thymidine synthesis. We observed a stronger effect on acceleration of Myc-induced lymphomagenesis in homozygous mutant *Shmt1* mice, suggesting that the salvage pathway is not completely penetrant. Taken together, we would argue that Shmt1 is a tumor-suppressing modifier in the context of B-cell lymphomas and colorectal adenomas.

One of the most important reasons for the systematic analysis of Myc target genes in tumorigenesis is the potential of identifying or validating future drug targets. Our lymphoma and adenoma data cast doubt on the utility of developing targeted interventions against Ldha, Phgdh and Shmt1. On the other hand, in these models, tumors arise in mice carrying germline mutations of both the oncogenic lesion and the genes encoding the metabolic enzymes. It is thus plausible that adaptations have occurred during development that would not have occurred in cells acutely exposed to an inhibitor. Nevertheless, our data suggest that tumor cells eventually will develop resistance to putative treatments directed against metabolic enzymes since tumor growth undoubtedly can occur in the absence of Shmt1, Phgdh or Ldha. Therefore, a correct stratification is needed to identify patients whose tumors would be sensitive to inhibitors that are under development, for instance against Ldh [6], [18]. Such stratification can be performed based on which oncogenic driver mutation the tumor has acquired. As shown here, oncogenic Ras or pathways utilizing this circuit could be a potential parameter. Moreover, two independent studies published while revising this manuscript suggest that *PHGDH* amplification could be another oncogenic driver mutation in breast cancer and melanoma, which would sensitize cells to inhibition of Phgdh [51], [52]. As shown here and in these two studies, Phgdh is important in some but not all contexts.

To date, very few inhibitors against Ldh have been identified and those known are either poorly bioavailable and/or have other targets. For instance, sodium oxamate is used in high millimolar concentrations but inhibits aspartate aminotransferase at concentrations where lactate production is not even affected when tumor cell growth is [53]. Gossypol, a natural compound from cottonseed first identified as a male contraceptive, also inhibits anti-apoptotic proteins of the Bcl-2 family making interpretation of anti-cancer activity difficult [54]. Even if improved inhibitors are developed the issue whether or not Ldh is a good target is unresolved. Our genetic study shows that cells carrying a defective Ldha are capable of forming tumors, albeit hindered by hypoxia. We and others also show that ablation of *Ldha* by RNAi can be detrimental for the cell. It is thus possible that either cell context determines sensitivity, or that the ablation of Ldha protein (RNAi) is more severe than inhibition of its activity (D223H mutation). This notion would lend support to the idea that Ldha may have other functions, potentially disconnected from its activity [55]. For instance, Ldha can be phosphorylated (Tyr238) and localized to the nucleus [56] and has recently been shown to exist in transcription complexes in ES cells [57]. Future studies should address if glycolysis-independent functions of Ldha, as suggested in transcription [58], [59], are the most important functions in some tumors. If so, focus on the development of new therapies should aim at blocking all activities of Ldha.

## Materials and Methods

### Ethics statement

All animal experiments were performed in accordance with the Regional Animal Ethic Committee Approval no. A6-08 or no. A18-08.

### Mouse colonies

All transgenic mice in the study were on pure C57BL/6 background. The λ-*Myc*-mice and the *Ldh* ^mut/mut^ mice have been previously described [24], [31]. The *p53* knockout mice and the *Apc* ^Min^ mice were from Jackson Labs, the C57BL/6 and Balb/c mice used as recipients were from Taconics, and the *Phgdh* knockout mice were from RIKEN BRC, Japan. *Shmt1* knockout mice were generated by blastocyst injection of gene-trapped ES cells (clone AD0236, Sanger Institute Gene-trap Resources) at Umeå Transgene Core Facility. After confirmation of germline transmission, mice were backcrossed to C57BL/6 for at least ten generations. Illumina SNP genotyping confirmed that the mice were at least 96% C57BL/6 before starting interbreeding with λ-*Myc* transgenic mice, *p53* knockout mice and *Apc* ^Min^ mice.

### Tumor monitoring and analyses

All mice used in the study were monitored by group members and personnel at the animal facilities (Umeå Transgene Core Facility or Helmholtz Centre, Munich). When showing signs of disease, λ-*Myc* mice were sacrificed and lymphomas were collected for analyses. Dates of sacrifice were entered into GraphPad Prizm software for the generation of survival curves. Lymphomas were either snap frozen for RNA and protein analyses, or formalin-fixed and embedded in paraffin. Paraffin blocks were sectioned and processed by standard immunohistochemistry methodology using antibodies directed against smooth musle actin (SMA; Sigma) or CD34 (Abcam) at the Histocenter core facility (Göteborg, Sweden).

The *Apc* ^Min^ mice used for adenoma formation studies were sacrificed and analyzed between 120–140 days of age, or when showing signs of disease. The small intestine and colon were dissected out, washed with phosphate-buffered saline and cut length-wise at which point adenomas were counted and tissues were harvested for analyses. Adenomas were counted using dissection microscope as well as by eye by two independent observers. The adenomas were scored irrespective of size and numbers per mouse were entered into GraphPad Prizm software for generation of graphs.

### Microarray analysis

The analysis of gene expression changes between magnetically sorted B cells from wildtype or λ-*Myc* transgenic mice was performed using the Illumina BeadChip system. For in vitro transcription amplification, 200 ng of RNA was used with the Illumina RNA Amplification Kit (Ambion). Amplified RNA (1.5 µg) was hybridized to the Sentrix MouseRef-8 Expression Beadchip (Illumina). The primary data were collected from the BeadChips using the manufacturer's BeadArray Reader and analyzed using the supplied scanner software. Data normalization was performed by cubic spline normalization using Illumina's Beadstudio v3 software. Clustering and visualization of genes encoding metabolic enzymes was done using the Spotfire software.

### Cell culture

MEFs were generated by mechanical disruption and trypsin-digestion of E13.5 embryos from which the fetal liver and the head had been removed. The single-cell suspension was grown in DMEM supplemented with 10% fetal bovine serum (FBS), 50 µM β-mercaptoethanol, 1× glutamine, pyruvate, non-essential amino acids and antibiotics (Invitrogen). 293T cells and NIH 3T3 (from ATCC) were routinely maintained in DMEM supplemented with 10% FBS, glutamine and antibiotics. Colon 26 cells (from Cell Line Services) were cultured in RPMI supplemented with 10% FBS, glutamine and antibiotics. The λ820 cell line was established from λ-*Myc* transgenic mice and cultured as previously described [38].

### Viral production and transductions

Retroviruses and lentiviruses were produced by calcium phosphate-mediated transfection of 293T cells. For retroviruses the following plasmids from Addgene were used: pBABE-HrasG12V-puro, pBABE-HrasG12V-hygro, MSCV-*Myc*-IRES-GFP, pWZL-Myc-blasticidine, pBABE-puro, pBABE-hygro together with pCL-Eco (encoding gag, pol and ecotropic envelope). Lentiviruses for RNAi were made using pLKO.1 puro vectors expressing shRNAs (Sigma Mission RNAi), together with packaging plasmids pCMV R8.2dvpr and pHCMV-EcoEnv (both from Addgene). Two or three different shRNAs were used per gene (Table S2) and they were compared to a control pLKO vector expressing a control shRNA with no known target in the mouse genome (non-target vector from Sigma). Thirty-six hours post-transfection, the media was harvested four times during an additional 36 h. The virus was filtered and either frozen down in aliquots or applied on target cells in the presence of 4–8 µg/ml polybrene. Following antibiotics selection for 48 h (or GFP analysis of FACS to confirm at least 90% positive cells) cells were expanded and used for experiments. The shRNA-containing viruses were always introduced into NIH 3T3 cells after the transduction with control, Myc or Ras retroviruses.

### Subcutaneous tumor formation and analyses

MEFs used for sarcoma formation studies were infected with retroviruses encoding oncogenic Hras, whereas Colon 26 cells were infected with lentiviruses expressing shRNA against *Shmt2*, *Phgdh* and *Ldha*. For MEFs, 1×10^6^ were injected subcutaneously into C57BL/6 recipients, whereas 5×10^5^ Colon 26 cells were mixed with Matrigel (1∶1) and injected into Balb/c mice. When tumors appeared, the mice were sacrificed and tumors were weighed and material was harvested for analyses. For immunofluorescence, formalin-fixed tumors were embedded in paraffin and sectioned (8 µm) onto glass slides. Following deparaffinization and rehydration, slides were either stained with H&E or subjected to Hoechst and antibody staining using Cy3-conjugated control or anti-smooth muscle actin antibody (Sigma) according to standard methodology. Following mounting the sections they were analyzed in a fluorescence microscope.

### Fetal liver transplants

Fetal livers of E13.5 embryos were dissected out of embryos from timed pregnancies between λ-*Myc*; *Phgdh* ^+/−^ males and *Phgdh* ^+/−^ females. Each individual liver was dissociated through a cell strainer and injected via the tail-vein into one lethally gamma-irradiated (9.25 Gy) C57BL/6 recipient. Tissue from each embryo was taken for genotyping and the mice positive for the λ-*Myc* transgene were followed for lymphoma development and treated as previously described in *Mouse colonies* and *Tumor monitoring and analyses*.

### Protein and RNA expression

For protein expression analyses by Western blot, cells and tumors were lysed in an appropriate amount of lysis buffer on ice for 30 min. Following sonication, clearing by centrifugation and protein determination, an equal amount of protein per well was loaded on SDS-PAGE gels and separated by electrophoresis. The proteins were transferred to a nitrocellulose membrane, which was subsequently blocked with TBST containing 5% non-fat dried milk. The membranes were then blotted with primary and horseradish peroxidise-conjugated secondary antibodies dissolved in blocking solution. After washing with TBST, the bound proteins were visualized by enhanced chemoluminescence. The primary antibodies used were from BD Biosciences (H-Ras), Cell signalling (c-Myc and Pdk1), Sigma-Aldrich (Ldha, Ldhb, Shmt1, Shmt2 and β-actin) and Atlas Antibodies (Shmt1 and Phgdh).

RNA expression was measured by quantitative reverse transcriptase PCR (qRT-PCR). Briefly, RNA was prepared using the NucleoSpin RNA II kit (Macherey-Nagel). cDNA was prepared using the First strand synthesis kit (Fermentas) and the PCR was run using the KAPA mastermix (Biotools) on an iQ real-time PCR machine (Bio-Rad). Primer sequences can be found in Table S3.

### Hypoxia experiment

NIH 3T3 cells expressing either HrasV12, c-Myc or both were used for parallel infections of lentiviruses encoding shRNAs against *Ldha*. The same amount of cells were seeded in 24-well format and subsequently infected. 72 hours post infection, each well was split into 3×96 well format in duplicate plates. One set of plates was placed in a hypoxic environment (using the Modular incubator chamber, Billups-Rothenberg Inc.) for 40 hours after which the hypoxic treatment was terminated and 3H-thymidine was added to all wells. After two hours, the plates were freeze-thawed and the cells harvested onto glass fibre filters. Microscint scintillation solution was administered to the dried filter, which were subsequently counted on a TopCount scintillation counter. λ820 cells were subjected to hypoxia in 24 well plates or 25 cm^2^ flasks (2×10^5^ cells/ml) and were harvested for cell counting and apoptosis analyses or RNA analyses, respectively. For apoptosis analyses, cells were stained with Vindelövs reagent (10 mM Tris, 10 mM NaCl, 75 µM propidium iodine, 0.1% Igepal, and 700 units/liter RNase adjusted to pH 8.0) and then analyzed with a FACScalibur flow cytometer (BD Biosciences). Apoptosis was determined using DNA histograms and was based on the number of cells that carried less than diploid DNA content (sub-G1) in a logarithmic FL2 channel.

### Enzyme activity assay

Total protein lysates were prepared as described above and the same amount of protein was assayed for LDH activity using the Cytotoxicity detection kit (Roche Applied Science). The reactions were read using the Tecan Infinite200 plate reader at 492 nm. Lysates were also used to assess Shmt activity and Phgdh in accordance with published methods [60], [61].

## Supporting Information

Figure S1 Metabolic pathways linking glycolysis and serine/folate metabolism. The enzymes: LDH (lactate dehydrogenase); PHGDH (3-phosphoglyerate dehydrogenase); PSAT (phosphoserine aminotransferase); PSPH (phosphoserine phosphotase); SHMT (serine hydroxymethyltransferase 1 and 2); DHFR (Dihydrofolate reductase); TYMS (Thymidylate synthase).
(PDF)

Figure S2 Further characterization of *Shmt1* mutant mice. (A) Protein expression of Shmt1, Shmt2 and Phgdh in λ-*Myc* cells and tumors. (B) Shmt activity in B cells from three 4-weeks old λ-*Myc* and three wildtype littermates. (C) Genomic organisation of the mouse *Shmt1* locus indicating insertion of the gene-trap cassette. (D) Typical PCR genotyping results of tail DNA from offspring of matings between heterozygous *Shmt1* mutant mice. (E) Western blot analysis showing absence of detectable protein in tissues from *Shmt1* mutant mice.
(PDF)

Figure S3 Ldha is essential for Colon 26 cells. (A) Subcutaneous tumors arising in syngenic Balb/c mice by injecting Colon 26 cells infected with lentiviruses expressing shRNAs against *Shmt2* and *Phgdh* (three separate hairpins of each). The symbols correspond to the different hairpins used in the experiment. In the control group, black symbol represents uninfected (UI) cells and white refers to the control non-targeting shRNA (NT). In the *Phgdh* shRNA group, white symbol represents shRNA#2, grey symbol shRNA#3 and black symbol shRNA#5. In the *Shmt2* group, white symbol represents shRNA#3, grey symbol shRNA#4 and black symbol shRNA#5. See Table S2 for details on the specific shRNA constructs. * Colon 26 cells expressing *Ldha* shRNA#2 and shRNA#5 were depleted in culture and could not be transplanted (B) The level of knockdown in the tumors from panel C was analyzed by qRT-PCR. (C) Colon 26 cells were transiently co-transfected in 6-well plates with a GFP-expressing plasmid and a plasmid expressing either a non-targeting (NT) shRNA or *Ldha* shRNA#5. Cells were analyzed for GFP and found to be 50–60% GFP-positive 24 h post-transfection (data not shown). At 72 h post-transfection, cells were counted. Shown is the mean ± SD of three independent experiments. *p<0.05 (D) The same cells as in C were analyzed by qRT-PCR to confirm *Ldha* knockdown.
(PDF)

Figure S4 Further characterization of *Ldha* mutant mice. (A) Splenomegaly in *Ldha* mutant mice. (B) Ldh activity in magnetically sorted splenic B cells derived from mice of indicated genotypes. (C) Survival curve of λ-*Myc* mice generated from interbreedings between *Ldha* ^mut/mut^ and λ-*Myc* transgenic mice generated at Helmholtz Center in Munich. (D) Ldh activity in some of the tumors developed in mice described in Figure 4A. (E) Western blot analysis of Ldha and Ldhb in some of the tumors developed in mice described in Figure 4A and 4B.
(PDF)

Figure S5 Hypoxia sensitizes fibroblasts cells to Ldha inhibition. (A) Immunofluorescence analysis of α-Smooth muscle actin in representative sarcomas from Figure 4D. Hoechst staining was used to stain the nuclei. (B) Western blot analysis for Ha-Ras on representative sarcomas from Figure 4D. (C) NIH 3T3 cells were infected with Myc, Ras or Myc+Ras retroviruses, followed by lentiviral infection with a control or an *Ldha*-targeting shRNA. The cells were subjected to hypoxia and cells were either analyzed by Western blot using antibodies against Ldha or Pdk1 (Hif target) (E) or incubated with radiolabelled thymidine to measure cell proliferation.
(PDF)

Figure S6 Myc-induced lymphoma cells are sensitive to hypoxia. (A–B) λ820 cells infected with lentiviruses expressing a non-target control shRNA (NT) or shRNAs against *Ldha* were subjected to hypoxic conditions. Quantification of apoptosis was performed by measuring the sub-G1 content using the gate shown in A and cell numbers were collected by counting viable cells. (C) qRT-PCR analysis for *Ldha* and *Pdk1* in the cells analyzed in B. (D) λ820 cells carrying an shRNA against *Ldha* or a control shRNA were transplanted into syngenic C57BL/6 recipients via the tail vein and monitored for tumor growth. (E) Ldh activities of λ820 cells infected with indicated lentiviruses.
(PDF)

Figure S7 Nodal lymphomas from λ-*Myc* transgenic mice express less angiogenic markers than splenic lymphomas. A) Paraffin-embedded lymphomas developed in lymph nodes of λ-*Myc* or λ-*Myc*;*Ldha^mut^* ^/mut^ mice were sectioned and stained with antibodies directed against angiogenic markers CD34 (brown staining) and SMA (red staining). Shown is one representative field of view encompassing most of the lymphoma (40×) or a larger magnification of the dashed square (200×). B) Paraffin-embedded spleens from wildtype mice or lymphomas developed in spleens of λ-*Myc* or λ-*Myc*;*Ldha^mut^* ^/mut^ mice were sectioned and stained with antibodies directed against angiogenic markers CD34 (brown staining) and SMA (red staining). Shown is one representative field of view at 200×.
(PDF)

Table S1 Expression of genes used in the unsupervised hierarchical clustering shown in Figure 1. Gene list is sorted based on the average fold-change between RNA expression in wildtype (wt) and λ-*Myc* splenic B cells.
(XLSX)

Table S2 List of shRNAs used in the study. The shRNAs came from the TRC1.0 mouse lentiviral library and their library number is indicated.
(XLSX)

Table S3 Sequences of qRT-PCR primers used in the study. Primers were designed using the SciTools at www.idtdna.com.
(XLSX)
