# Supplementary figures and images for: Mouse Genetics Suggests Cell-Context Dependency for Myc-Regulated Metabolic Enzymes during Tumorigenesis
Source: PLoS Genet. 2012 Mar 15;8(3):e1002573. doi: 10.1371/journal.pgen.1002573 (PMC3305401; doi:10.1371/journal.pgen.1002573)

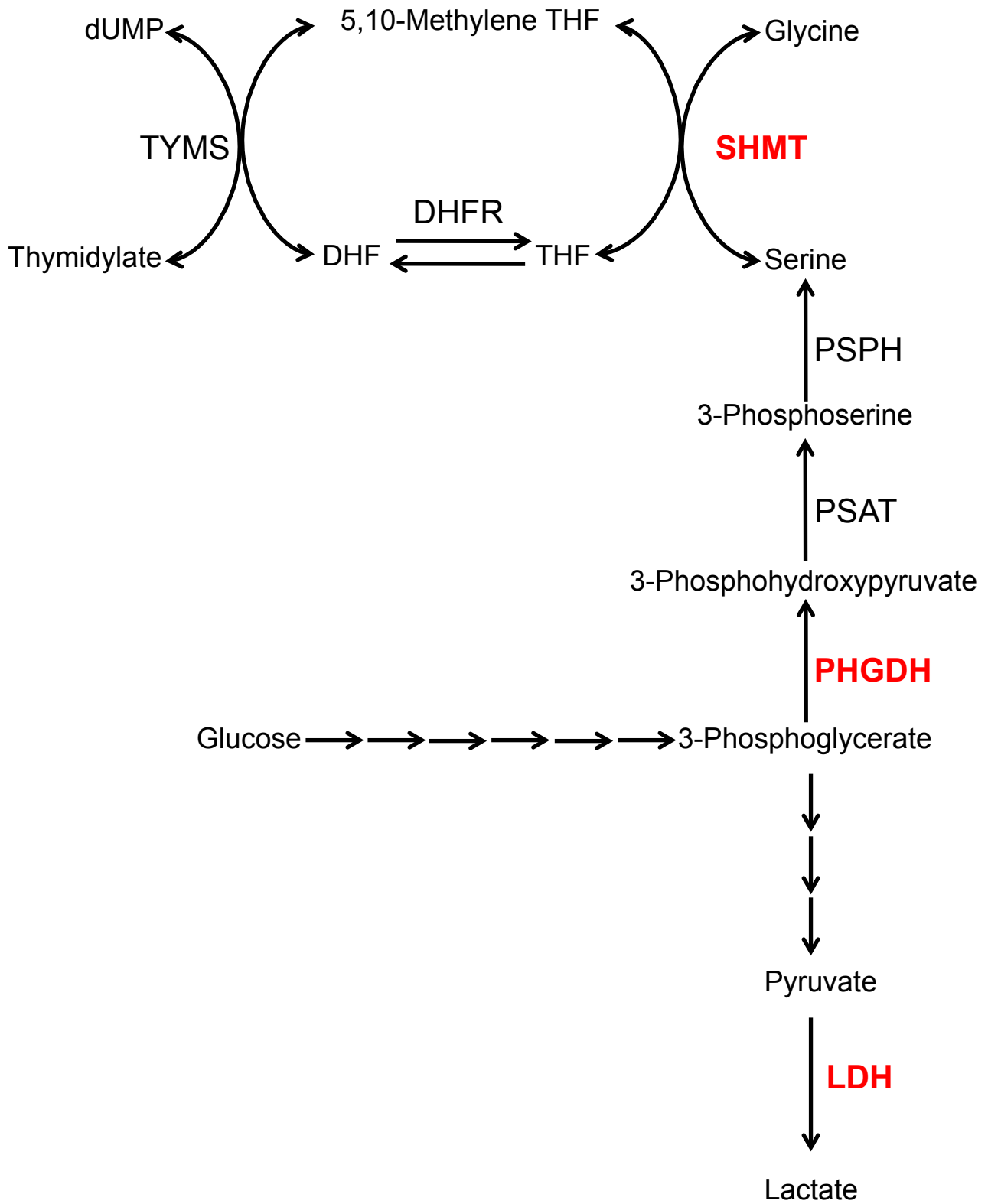

Supplement: Figure S1 — Metabolic pathways linking glycolysis and serine/folate metabolism. The enzymes: LDH (lactate dehydrogenase); PHGDH (3-phosphoglyerate dehydrogenase); PSAT (phosphoserine aminotransferase); PSPH (phosphoserine phosphotase); SHMT (serine hydroxymethyltransferase 1 and 2); DHFR (Dihydrofolate reductase); TYMS (Thymidylate synthase). (PDF) [file pgen.1002573.s001.pdf]

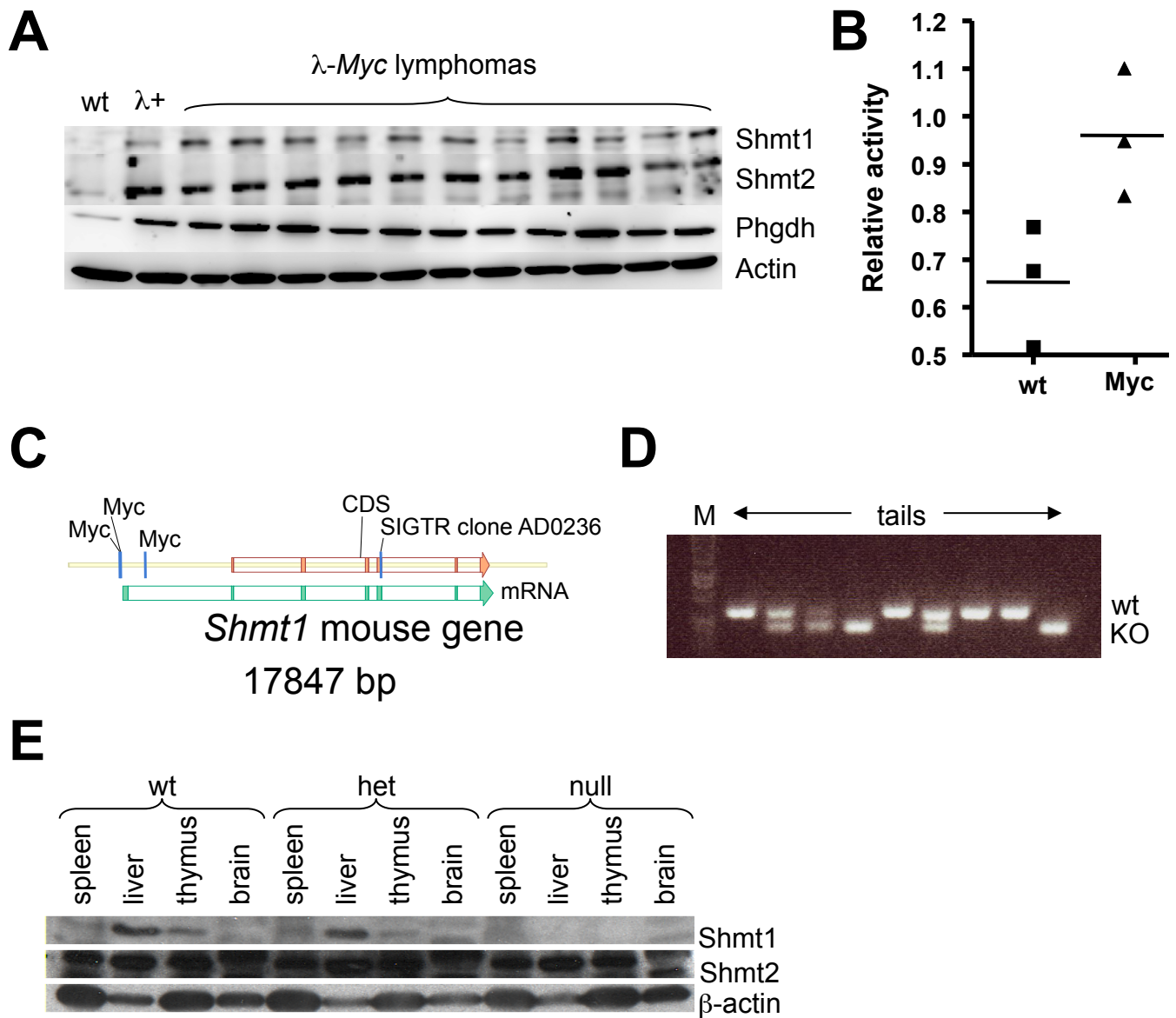

Supplement: Figure S2 — Further characterization of Shmt1 mutant mice. (A) Protein expression of Shmt1, Shmt2 and Phgdh in λ-Myc cells and tumors. (B) Shmt activity in B cells from three 4-weeks old λ-Myc and three wildtype littermates. (C) Genomic organisation of the mouse Shmt1 locus indicating insertion of the gene-trap cassette. (D) Typical PCR genotyping results of tail DNA from offspring of matings between heterozygous Shmt1 mutant mice. (E) Western blot analysis showing absence of detectable protein in tissues from Shmt1 mutant mice. (PDF) [file pgen.1002573.s002.pdf]

**A**

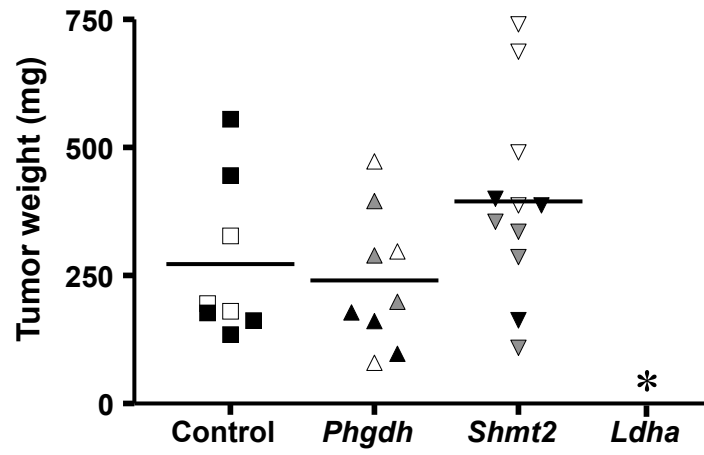

**B**

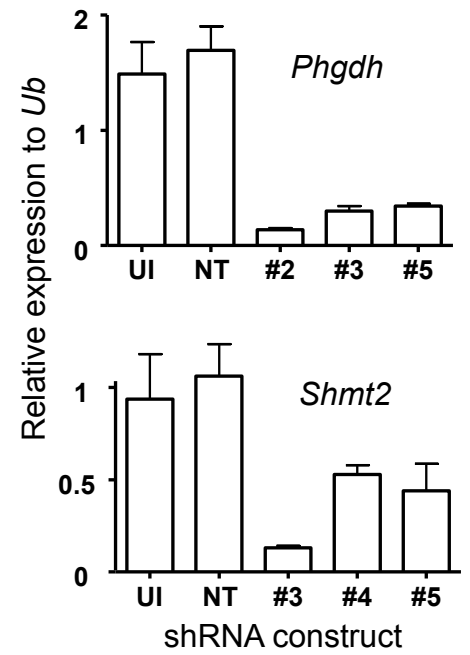

**C**

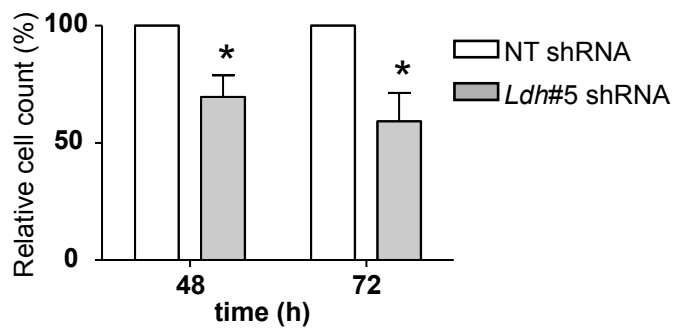

**D**

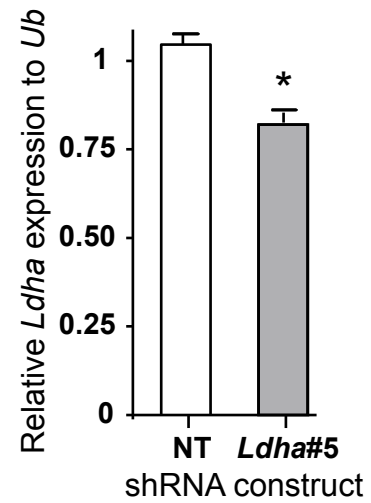

Supplement: Figure S3 — Ldha is essential for Colon 26 cells. (A) Subcutaneous tumors arising in syngenic Balb/c mice by injecting Colon 26 cells infected with lentiviruses expressing shRNAs against Shmt2 and Phgdh (three separate hairpins of each). The symbols correspond to the different hairpins used in the experiment. In the control group, black symbol represents uninfected (UI) cells and white refers to the control non-targeting shRNA (NT). In the Phgdh shRNA group, white symbol represents shRNA#2, grey symbol shRNA#3 and black symbol shRNA#5. In the Shmt2 group, white symbol represents shRNA#3, grey symbol shRNA#4 and black symbol shRNA#5. See Table S2 for details on the specific shRNA constructs. * Colon 26 cells expressing Ldha shRNA#2 and shRNA#5 were depleted in culture and could not be transplanted (B) The level of knockdown in the tumors from panel C was analyzed by qRT-PCR. (C) Colon 26 cells were transiently co-transfected in 6-well plates with a GFP-expressing plasmid and a plasmid expressing either a non-targeting (NT) shRNA or Ldha shRNA#5. Cells were analyzed for GFP and found to be 50–60% GFP-positive 24 h post-transfection (data not shown). At 72 h post-transfection, cells were counted. Shown is the mean ± SD of three independent experiments. *p<0.05 (D) The same cells as in C were analyzed by qRT-PCR to confirm Ldha knockdown. (PDF) [file pgen.1002573.s003.pdf]

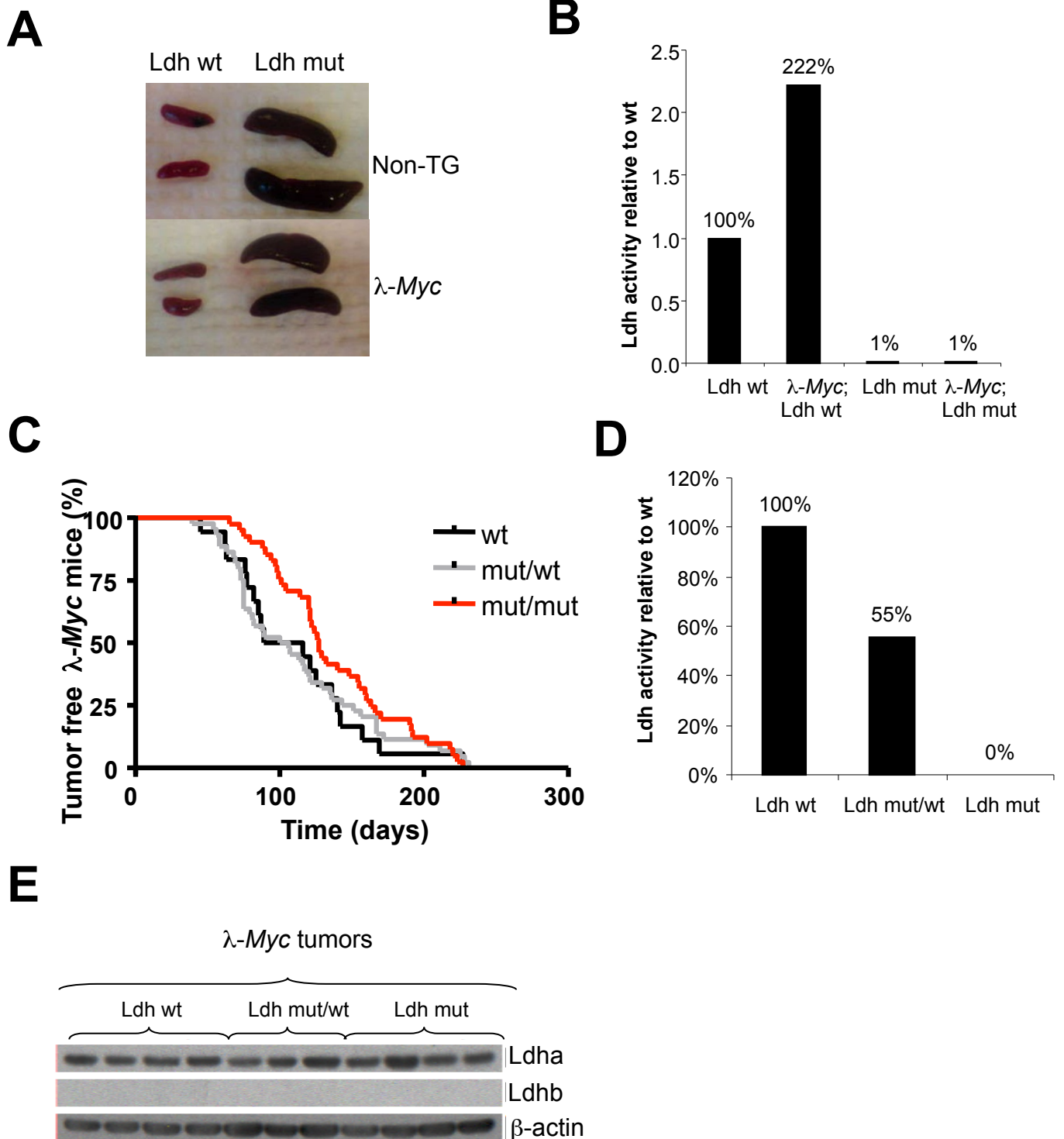

Supplement: Figure S4 — Further characterization of Ldha mutant mice. (A) Splenomegaly in Ldha mutant mice. (B) Ldh activity in magnetically sorted splenic B cells derived from mice of indicated genotypes. (C) Survival curve of λ-Myc mice generated from interbreedings between Ldha mut/mut and λ-Myc transgenic mice generated at Helmholtz Center in Munich. (D) Ldh activity in some of the tumors developed in mice described in Figure 4A. (E) Western blot analysis of Ldha and Ldhb in some of the tumors developed in mice described in Figure 4A and 4B. (PDF) [file pgen.1002573.s004.pdf]

**A**

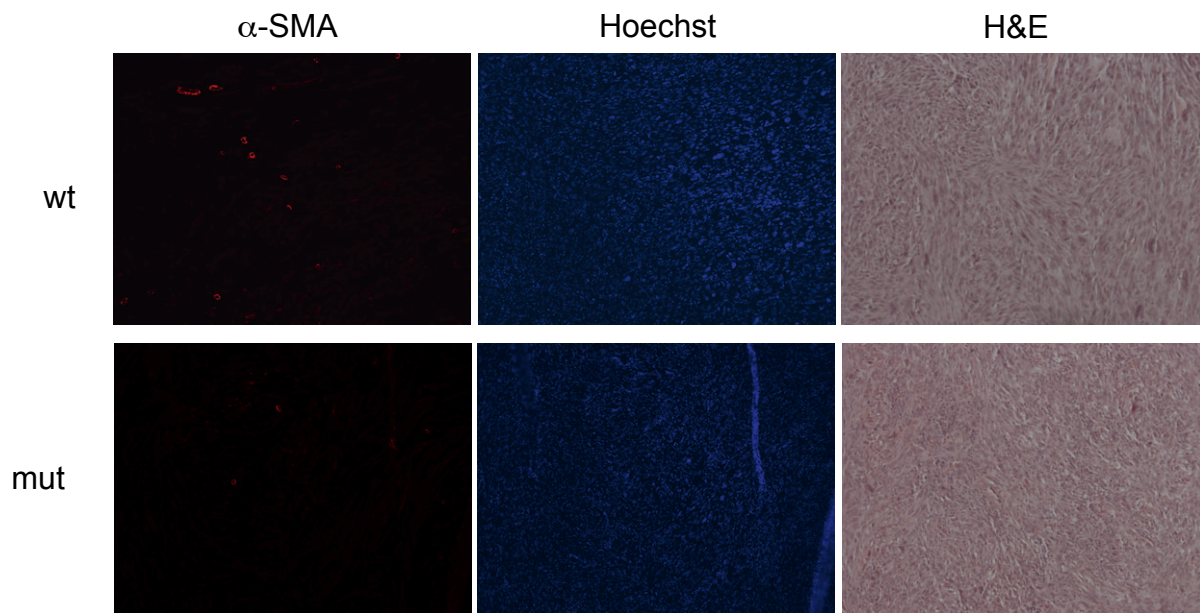

**B**

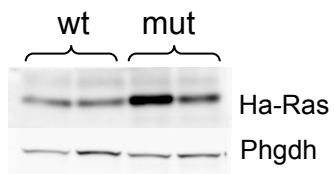

**C**

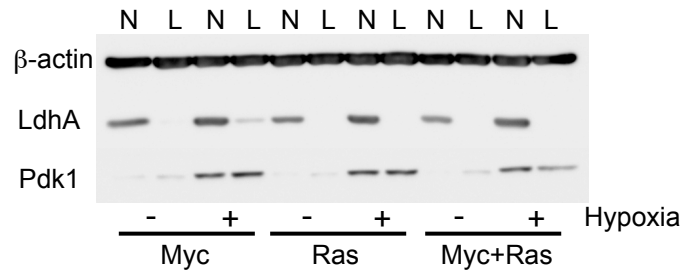

**D**

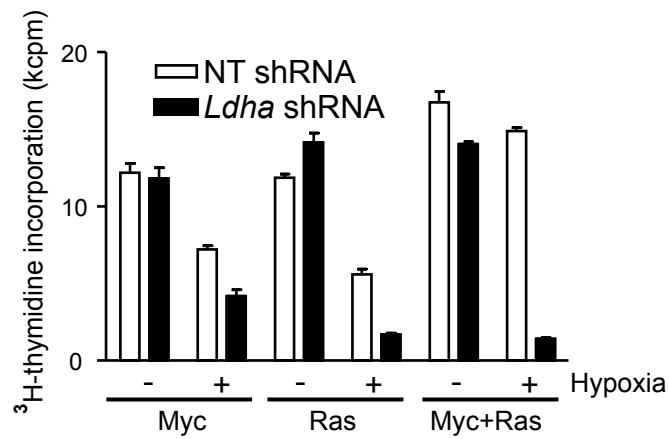

Supplement: Figure S5 — Hypoxia sensitizes fibroblasts cells to Ldha inhibition. (A) Immunofluorescence analysis of α-Smooth muscle actin in representative sarcomas from Figure 4D. Hoechst staining was used to stain the nuclei. (B) Western blot analysis for Ha-Ras on representative sarcomas from Figure 4D. (C) NIH 3T3 cells were infected with Myc, Ras or Myc+Ras retroviruses, followed by lentiviral infection with a control or an Ldha-targeting shRNA. The cells were subjected to hypoxia and cells were either analyzed by Western blot using antibodies against Ldha or Pdk1 (Hif target) (E) or incubated with radiolabelled thymidine to measure cell proliferation. (PDF) [file pgen.1002573.s005.pdf]

**A**

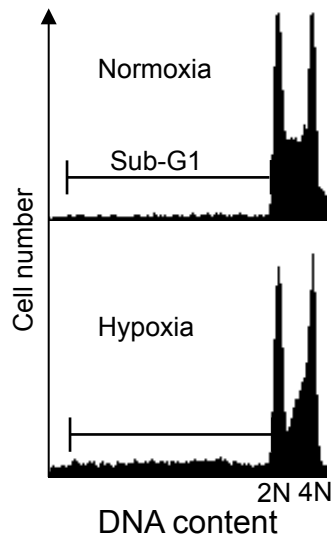

**B**

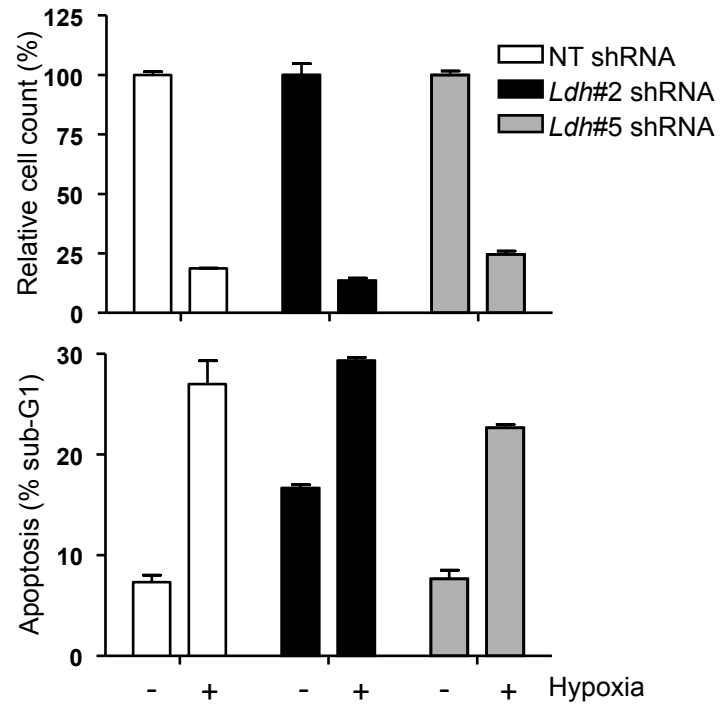

**C**

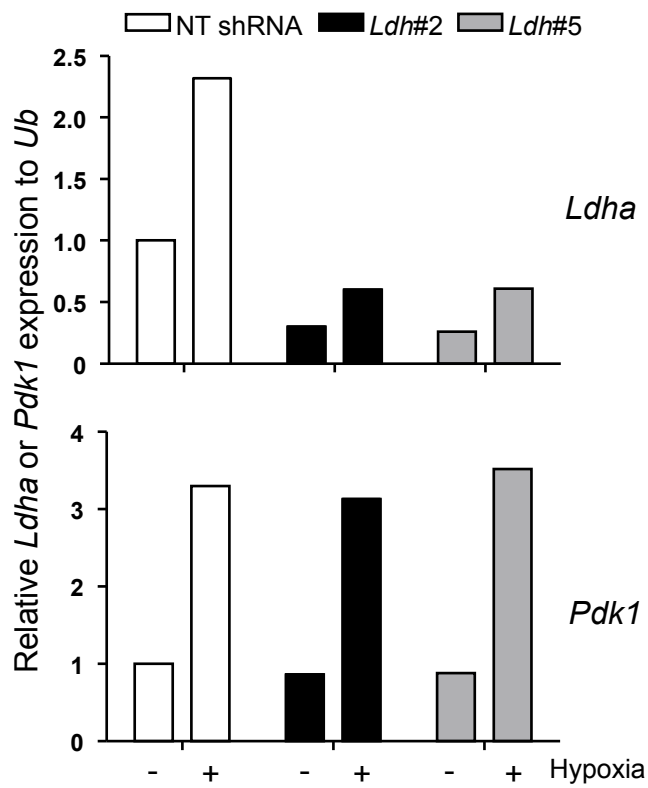

**D**

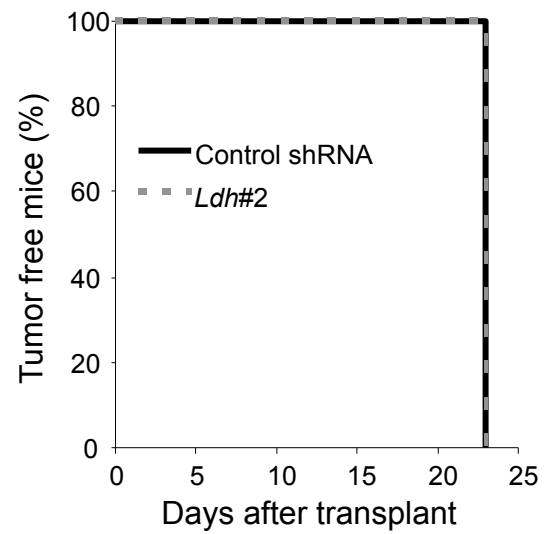

**E**

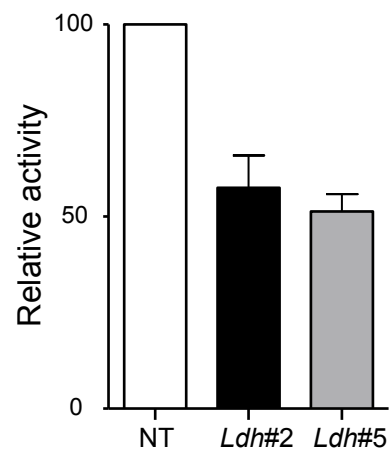

Supplement: Figure S6 — Myc-induced lymphoma cells are sensitive to hypoxia. (A–B) λ820 cells infected with lentiviruses expressing a non-target control shRNA (NT) or shRNAs against Ldha were subjected to hypoxic conditions. Quantification of apoptosis was performed by measuring the sub-G1 content using the gate shown in A and cell numbers were collected by counting viable cells. (C) qRT-PCR analysis for Ldha and Pdk1 in the cells analyzed in B. (D) λ820 cells carrying an shRNA against Ldha or a control shRNA were transplanted into syngenic C57BL/6 recipients via the tail vein and monitored for tumor growth. (E) Ldh activities of λ820 cells infected with indicated lentiviruses. (PDF) [file pgen.1002573.s006.pdf]

**A**

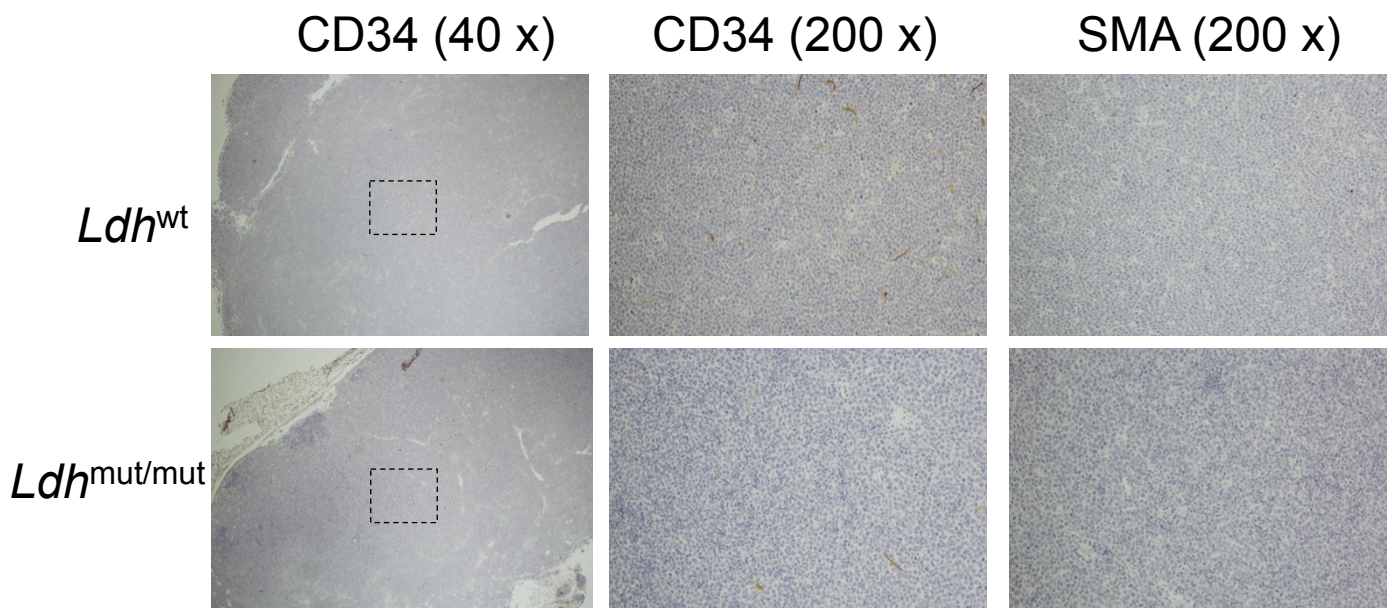

**B**

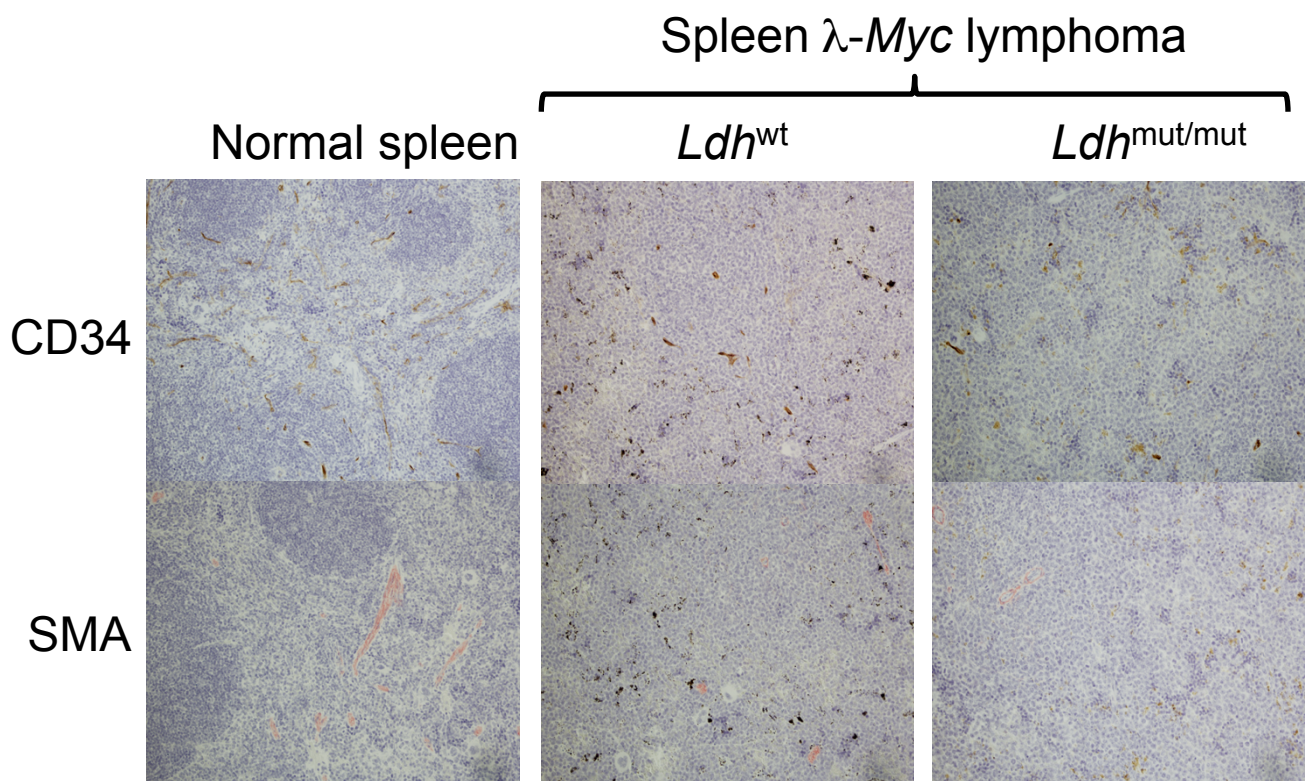

Supplement: Figure S7 — Nodal lymphomas from λ-Myc transgenic mice express less angiogenic markers than splenic lymphomas. A) Paraffin-embedded lymphomas developed in lymph nodes of λ-Myc or λ-Myc;Ldhamut /mut mice were sectioned and stained with antibodies directed against angiogenic markers CD34 (brown staining) and SMA (red staining). Shown is one representative field of view encompassing most of the lymphoma (40×) or a larger magnification of the dashed square (200×). B) Paraffin-embedded spleens from wildtype mice or lymphomas developed in spleens of λ-Myc or λ-Myc;Ldhamut /mut mice were sectioned and stained with antibodies directed against angiogenic markers CD34 (brown staining) and SMA (red staining). Shown is one representative field of view at 200×. (PDF) [file pgen.1002573.s007.pdf]
